# Perception of Periodontitis Patients about Treatment Outcomes: A Cross-Sectional Study in Saudi Arabia

**DOI:** 10.3390/healthcare12131288

**Published:** 2024-06-27

**Authors:** Khalid Saad Alkhurayji, Hessah Al Suwaidan, Farah Kalagi, Mohamed Al Essa, Mohammed Alsubaie, Saja Alrayes, Arwa Althumairi

**Affiliations:** 1 Health Information Management and Technology Department, College of Public Health, Imam Abdulrahman bin Faisal University, Dammam 34212, Saudi Arabia; 2240700039@iau.edu.sa (H.A.S.); 2240700037@iau.edu.sa (F.K.); salrayes@iau.edu.sa (S.A.); aalthumairi@iau.edu.sa (A.A.); 2 Dental Center, Prince Sultan Military Medical City, Riyadh 11159, Saudi Arabia; maalessa@psmmc.med.sa (M.A.E.); mfsubaie@psmmc.med.sa (M.A.)

**Keywords:** patient-centered, patient experience, periodontitis, treatment outcome

## Abstract

Patient compliance following periodontal therapy is extremely important in predicting the prognosis of the disease and maintaining treatment outcomes. Therefore, this study aimed to investigate the perception of periodontitis patients about treatment outcomes. A cross-sectional study was conducted among periodontitis patients in a single dental center through a pre-validated questionnaire that was distributed to each participant in the waiting area of periodontal clinics by utilizing a convenience sampling technique. Median and interquartile ranges were used in addition to frequency and percentages. Bivariate analyses were performed using the Mann–Whitney U and Kruskal–Wallis test. Among the 300 male and female participants, the median score (interquartile range) of the current level of pain revealed that males experienced more pain than females, with a median score of 5 (0–7) for males and 4 (0–6) for women. However, the median (interquartile range) for desired and expected pain levels in both genders was 0 (0–1), 0 (0–4). There were significant differences in median score ratings between males and females for expected, distress, success, and importance levels (*p*-value < 0.05). Patients with periodontitis provided valuable insights into the experiences of individuals undergoing treatment for periodontal disease, indicating overall patient satisfaction with the expected levels of periodontal outcomes.

## 1. Introduction

Untreated gingival inflammation can further progress and lead to more advanced periodontal destruction and the loss of periodontal attachment and bone support. This destructive process is considered the foundation of an inflammatory disease called periodontitis [1]. One of the most significant criteria for the diagnosis of periodontitis is the measurement of clinical attachment loss (CAL) and the gingival probing depth (PD). A CAL of 2 mm and a 3 mm PD indicates periodontitis [2].

Multiple intraoral consequences could be the result of untreated periodontitis, which eventually leads to tooth loss, especially in the presence of other risk factors, such as smoking [3]. However, Schätzle, Löe [4] failed to relate the effects of a lifelong light smoking habit to tooth loss. Additional common complications experienced in the cases of periodontitis are discomfort and tooth sensitivity, arising from gingival recession with exposed root surfaces [5]. 

More seriously, several reports have argued the association between periodontitis and several systemic diseases, into which cardiovascular disease (CVD) complies with the main concerns [6]. Furthermore, diabetes mellitus (DM) has an intimate relationship with periodontitis in both directions, since DM can be considered one of the risk factors for the progression of periodontitis, and on the other hand, periodontitis can adversely affect the average blood glucose level (HbA1c) in patients with DM [7]. 

Different surgical and non surgical treatment options are available to arrest disease progression and maintain periodontal health [8]. Despite recent advances in periodontal treatment strategies, mechanical plaque control, and calculus removal through scaling and root planning remain the gold-standard management options [9]. Various surgical techniques, such as gingivectomy, flap debridement, and modified Widman flap can be added to the conventional non-surgical treatment methods to improve the outcomes in severe chronic periodontitis cases [10]. Antimicrobial therapy through the administration of systemic antibiotics can be considered an adjunctive to conventional treatment in the cases of persistent periodontitis, or even in certain forms of the disease, such as localized juvenile and aggressive adult periodontitis [11]. 

Oral health-related quality of life (OHRQoL) focuses on identifying the person-centered outcome within several dimensions and concepts [12]. According to Jaumet, Hamdi [13], the quality of life and well-being of periodontitis patients across sex and gender were found to be low. Considering improvements in these areas, periodontal disease therapy increases overall quality of life and allows patients to express themselves more about their treatment outcomes and expectations [14]. 

The importance of patients’ perception of treatment outcomes contributes to leveling patient satisfaction and expectations [15]. A wide variety of questionnaires were employed to assess the perception of treatment outcomes post periodontal surgeries or for oral impacts on daily performance [16,17]. According to a recent review of the tools used for measuring the outcome of periodontal therapy, patient-reported outcome (PRO) was used to collect information for periodontal treatment outcomes. PROs can be classified as generic or disease- and condition-specific. Presently, no consensus on a preferred tool for periodontal clinical research has been reached, and the selection of the questionnaire is to be related to familiarity with PRO in the research team. Additionally, the common PRO used needs to determine sufficient reliability and validity [18].

Compliance following periodontal therapy is extremely important in predicting disease prognosis and maintaining treatment outcomes [19]. Al Bush [20] confirmed that, among many factors, patient satisfaction regarding the received periodontal treatment strongly influences the level of postoperative compliance. Dirham [21], determined a high percentage of satisfaction for patients living in Saudi Arabia following non-surgical periodontal therapies. Further, a cross-sectional survey conducted in 2016 showed a higher level of patient satisfaction, with less pain experiences following non-surgical periodontal therapies in comparison to the surgical approaches to treatment [22]. Therefore, the study aimed to investigate the perception of periodontitis patients about the treatment outcomes at one point in time, to allow researchers to examine numerous characteristics at once.

## 2. Materials and Methods

### 2.1. Research Design

A cross-sectional design, to investigate the patient-centered outcome among periodontitis patients in a single dental center in Saudi Arabia from a primary source. 

### 2.2. Participants

Inclusion criteria: (i) patients diagnosed with periodontitis in their medical record and (ii) those who provided informed consent. Exclusion criteria: (i) patients who have not been officially diagnosed with periodontitis.

### 2.3. Sampling Procedures

The G power analysis program was used to calculate the sample size to be a minimum of 145 considering an effect size of 0.2 from a previous study [10], a power of 0.8, and a 0.05 alpha error probability [23]. A sample size of 300 patients represents a sufficient population size that can provide appropriate statistical power to spot the expected differences. A larger sample size improves the generalizability of the outcomes and increases the precision of outcomes. 

### 2.4. Instruments

A pre-validated patient-centered questionnaire was used (See Appendix A), which rates dimensions of importance for periodontitis patients in terms of interference, fatigue, pain, and distress on an 11-point scale from 0 to 10 [24,25]. This unique questionnaire evaluates current, important, successful, expected, and desired levels. However, the important level was answered from 10 to 0.

### 2.5. Data Collection

The data collection process started from July to August 2023. Questionnaire distribution held in the dental center was paper-based for each patient in the waiting area of the periodontal clinics, by utilizing a convenience sampling technique. 

### 2.6. Statistical Analysis

SPSS version 25 was used for descriptive analysis, and normality assumptions were not fulfilled. Therefore, the median and interquartile range were used in addition to frequency and percentages. Bivariate analyses were performed using the Mann–Whitney U test, and Kruskal–Wallis, and the statistical significance level was set at ≤0.05 for all estimations. Treatment outcomes in this study are the dependent variables, whereas patients’ perceptions of periodontitis were the independent variables.

### 2.7. Ethical Code

The researchers adhere to the ethical codes of health research. The researcher prioritized the protection of participants in this study. Additionally, informed consent was obtained from all participants, ensuring they understood the purpose of the study, voluntary participation, confidentiality of their responses, and the right to withdraw at any time. Furthermore, participants’ anonymity and confidentiality will be maintained, and all data will be securely stored and accessible only to the research team. The authors adhere to the institutional review board to ensure the study complies with ethical codes and standards for research conducted on human subjects.

## 3. Results

### 3.1. Demographic Data of the Participants

This study included 300 male and female participants, with the majority of them (61%) being male and encompassing the age range of 30 to 50 years old (50.7%). Nearly all of those who participated in the study (86%) were married. Furthermore, most participants (65.3%) were high school graduates, with a minority of participants (36%) responding no to chronic medical illness (Table 1).

### 3.2. Perceptions of Participant Characteristics and Treatment Outcomes

Table 2 depicts the outcomes of the participant’s characteristics and the pain score of the patient-centered outcome in terms of current, desired, expected, and important levels. The bivariate analysis showed significant differences between males and females in median score ratings for desired and successful levels (*p*-value < 0.05). However, no statistical significance was observed at the expected, current, or important levels (no difference between the two groups). Furthermore, the median score (interquartile range) of the current level of pain revealed that males experience more pain than females, with a median score of 5 (0–7) for males and 4 (0–6) for women. However, the median expected pain level in both genders was 0. Additionally, there were significant differences in median score ratings between participants with and without chronic illness, including current, desired, successful, expected, and important levels (*p*-value < 0.05). Furthermore, the median score (interquartile range) of the current level of pain revealed that participants with chronic disease scored 0 (0–5), whereas those without chronic illness had a median score of 6 (3–7). 

The results illustrate significant differences in median score ratings across age groups, including current, desired, successful, expected, and important levels (*p*-value < 0.05). Moreover, participants aged below 30 years had a median pain score of 10 (7–10). However, the median score (interquartile range) among those over 50 was 3 (0–5). Furthermore, there were significant differences in median score ratings among the different marital status groups, including current, desired, successful, expected, and important levels (*p*-value < 0.05). The median score (interquartile range) for current pain among married participants was 5 (0–6), lower than that of unmarried participants, which was 7 (3–10). However, the median score (interquartile range) for the important level for married participants was 10 (8–10), higher than that for single participants, which was 9 (9–10). 

In terms of education, there were statistically significant differences in median score ratings across education levels, including current, desired, successful, expected, and important levels (*p*-value < 0.05). The median score (interquartile range) of participants with a diploma was 0 (0–0). However, the median (interquartile range) of current pain among participants with a bachelor’s degree was 6 (3–9). The successful level varied between groups, with diploma holders and high school participants reporting the same median 0 (0–1), 0 (0–0). Higher education and bachelor’s degree holders had a median (interquartile range) score of 2 (0–2), 2 (2–2).

Table 3 shows no significant differences in median score ratings between males and females at any level (no difference between the two groups). Furthermore, the median score (interquartile range) of current fatigue levels revealed that women were more fatigued than men, with a median score of 2 (0–5) for women and 0 (0–8) for males. However, women indicated that improving treatment outcomes was important, with a median score (interquartile range) of 10 (9–10) among women and 10 (8–10) among men. 

In terms of chronic illness, there were significant differences in fatigue levels according to the median score ratings between participants, including current, desired, successful, expected, and important levels (*p*-value < 0.05). Furthermore, the median score (interquartile range) for the current fatigue level revealed that participants with chronic illness scored 0 (0–0). However, the participants without chronic illness had a median score (interquartile range) of 3 (0–8). Furthermore, the results indicate significant differences in median score ratings across education levels, marital status, and age groups (*p*-value < 0.05). Moreover, the median (interquartile range) of current fatigue among participants aged 30 years or less was 8 (6–8). However, participants aged 30 to 59 years had fatigue scores of 0 (0–4), whereas those over 50 had 0 (0–5).

Table 4 illustrates the findings of the participant’s characteristics, and the distress found significant differences in median score ratings between males and females for success, expected, and important levels (*p*-value < 0.05). However, current levels (*p*-value = 0.205) and expected levels (*p*-value = 0.980) levels did not significantly differ. 

The distress levels among participants with and without chronic illness showed significant differences in median score ratings, including desired, successful, expected, and important levels (*p*-value < 0.05). However, the current level did not significantly differ between the two groups (*p*-value = 0.491). Furthermore, participants with and without chronic illness reported a median score (interquartile range) of 5 (0–8), and the current level of distress revealed that both men and women reported the same median score of (interquartile range) of 5 (0–8). 

The distress levels revealed significant differences in median score ratings according to education level, marital status, and age group (*p* < 0.05). However, marital status and importance level did not show statistical significance (*p*-value = 0.093). Furthermore, the median (interquartile range) current distress score among participants aged below 30 years was determined to be 8 (8–8). However, those aged 30 to 50 years had a distress score of 4 (0–7), while those over 50 had a distress score of 5 (0–7). 

Table 5 illustrates the findings of the participant’s characteristics and interference levels’ score. The results showed significant differences in median score ratings between males and females for current, desired, and expected levels (*p*-value < 0.05). However, the successful (*p*-value = 0.768) and importance (*p*-value = 0.294) levels did not significantly differ. 

Participants with and without chronic illness exhibited significant differences in median score ratings of current, desired, successful, and expected levels (*p*-value < 0.005). However, the important level (*p*-value = 0.125) did not significantly differ between the two groups. Furthermore, the current level of interference revealed that participants with chronic illness reported a median score (interquartile range) of 5 (2–8). Additionally, the current level of interference had a median (interquartile range) score of 4 (0–8) for men and 5 (2–6) for women. 

Regarding education levels and interference in daily activities, the results showed significant differences in median score ratings across marital status and age groups (*p* < 0.05). However, the current level and marital status did not show any statistical significance (*p*-value = 0.066). Furthermore, the median (interquartile range) current distress score among participants aged 30 years or less was 5 (0–7). However, participants aged 30 to 50 years had an interference score of 3 (0–5), whereas those over 50 had a score of 5 (0–8).

## 4. Discussion

The current study focused on measuring patient-related outcomes among patients with periodontitis in Saudi Arabia. Most of the respondents were males between the ages of 30 and 50. This demographic distribution reflects the higher incidence of periodontitis in the middle-aged population and supports the representativeness of the sample [26,27]. In addition, most participants were married, which reflects a high level of responsibility and motivation to seek dental care [28]. For the educational background, the patients of the study were high school graduates, which implies that education level may influence the perception and understanding of periodontitis treatment outcomes. When discussing treatment choices and expectations with patients, healthcare practitioners need to pay special attention to their level of education to support treatment outcomes [29]. The study findings reflected that the patients mentioned current, desired, and expected levels of periodontitis outcomes as successful. This indicates that the patients were happy with the results of the treatment they received, and this also suggests that the interventions provided were able to address the patients’ periodontal problems [30]. Nevertheless, the median pain and distress were at a moderate level, which suggests that these factors require more attention during the treatment process [31].

Patients’ expectations, desires, and important levels strongly influence their perceptions of treatment outcomes, which is consistent with a previous study conducted by Al Bush [20], which found that, among other factors, patient satisfaction with the periodontal treatment received strongly influences the compliance gained postoperatively. These findings indicate that patient-centered outcomes emphasize offering the desired care for patients over the healthcare providers’ or institutions’ expectations of treatment outcomes. In the same context, Dirham, 2021 [21] reported a high degree of satisfaction among patients in Saudi Arabia after non-surgical periodontal therapy, which is compatible with the current study. As a result, among these levels, the expectation of treatment outcome includes a decline in pain, distress, fatigue, and interference with daily activities. The reason for this could be attributed to the severity of periodontitis. Furthermore, Shah, Xu [19] and Al Bush [20] found that a patient’s pain during periodontal therapy influences treatment outcome. However, Gautam, Galgali, and Mishra [22] revealed that patients experienced less pain and a higher level of satisfaction, which is consistent with this study’s findings. This finding may be related to the type of treatment received. In addition, medicine that serves to treat other conditions could play a major role.

There were significant differences between male and female respondents regarding expected distress, success, and importance levels. This suggests that there are gender disparities in perceptions of distress, success, and the importance of treatment outcomes among patients with periodontitis in Saudi Arabia. The observed results may be influenced by the male-to-female ratio, as the predominance of male participants (61%) could have skewed the findings toward experiences and perceptions more common among men. Almoudi, Hussein Almoudi, Hussein [32] suggested that cultural and gender differences can influence the perceptions of dental treatment outcomes, with females often reporting higher levels of distress and placing greater importance on successful outcomes compared to males. However, no significant differences between males and females were found in current and desired levels of distress, success, importance, and pain levels. This finding contrasts with previous research by Rekhi, Marya Rekhi, Marya [33], which reported gender differences in desired treatment outcomes among periodontitis patients. However, further investigation is needed to explore the factors contributing to this discrepancy.

The study also revealed significant differences in pain levels between male and female patients, particularly in successful and desired pain levels. This indicates that gender may play a role in the perception and experience of pain associated with periodontal treatment. Al-Khabbaz, Al-Shammari and Al-Saleh [34] reported that hormonal differences between males and females can influence pain perception and response to dental procedures.

The heterogeneity of the sample within the cross-sectional design is prone to sampling bias. However, this study used a larger sample to reduce biases in the results. In addition, several chronic illnesses and demographics have been revealed to be associated with periodontitis [35,36,37]. Therefore, the characteristics of the study sample were represented among the patient-centered outcome levels.

The limitation of this study was that the statistical significance could possibly attributed to large disparities in samples rather than actual differences. Future studies in this field should overcome this constraint and investigate perceptions of periodontal disease treatment outcomes among patients across different contexts for the generalizability of the results.

Further research should consider a larger and more diverse sample, and extend the scope of the present study by addressing the long-term effects of facilitating perspective-taking. The implications of our results for clinical practice underscore the role of the patients’ perception of their situation in affecting periodontal treatment planning.

## 5. Conclusions

The study provides valuable insights into the perceptions of periodontitis patients in Saudi Arabia, highlighting overall satisfaction with treatment outcomes. The predominantly middle-aged male sample and significant gender differences suggest cultural and physiological influences on experiences. Moderate pain and distress levels indicate areas that require more attention. The findings underscore the importance of tailored, patient-centered care for periodontal treatment.

## Figures and Tables

**Table 1:** Demographic data of the study’s participants.

Variable	N	%
Gender		
Male	183	61.0
Female	117	39.0
	300	100.0
Age		
Less Than 30 Years Old	42	14.0
From 30 To 50 Years	152	50.7
More Than 50 Years	106	35.3
	300	100.0
Marital Status		
Married	258	86.0
Single	32	10.7
Widow	10	3.3
	300	100.0
Education		
High School	196	65.3
Bachelor	72	24.0
Higher Education	10	3.3
Diploma	22	7.3
	300	100.0
Chronic Illness		
Yes	110	36.7
No	190	63.3
	300	100.0

**Table 2:** Descriptive statistics of pain levels with participant characteristics.

Participant Characteristics	Current Levels				Desired Levels				Successful Levels				Expected Levels				Importance Levels			
	Median	Q1	Q2	*p*-Value	Median	Q1	Q2	*p*-Value	Median	Q1	Q2	*p*-Value	Median	Q1	Q2	*p*-Value	Median	Q1	Q2	*p*-Value
Gender																				
Male	5	0	7	0.886	0	0	1	0.018 *	0	0	1	0.023 *	0	0	4	0.935	10	8	10	0.419
Female	4	0	6		0	0	0		0	0	2		0	0	3		10	9	10	
Age																				
Less Than 30 Years Old	10	7	10	0.000 *	5	1	5	0.000 *	2	0	2	0.000 *	4	4	10	0.000 *	9	9	10	0.026 *
From 30 To 50 Years	4	0	6		0	0	0		0	0	2		0	0	2		10	8	10	
More Than 50 Years	3	0	5		0	0	0		0	0	0		0	0	0		10	8	10	
Martial Status																				
Married	5	0	6	0.000 *	0	0	0	0.000 *	0	0	1	0.000 *	0	0	0	0.000 *	10	8	10	0.000 *
Single	7	3	10		1	0	5		2	0	2		4	0	10		9	9	10	
Widow	0	0	0		3	3	3		3	3	3		4	4	4		7	7	7	
Education Level																				
High School	5	0	7	0.000 *	0	0	0	0.000 *	0	0	1	0.000 *	0	0	4	0.000 *	10	8	10	0.000 *
Bachelor	6	3	9		0	0	6		2	0	2		0	0	5		9	8	10	
Higher Education	3	3	3		2	2	2		2	2	2		0	0	0		10	10	10	
Diploma	0	0	0		0	0	0		0	0	0		0	0	0		10	10	10	
Chronic Illness																				
Yes	0	0	5	0.000 *	0	0	0	0.000 *	0	0	0	0.000 *	0	0	0	0.000 *	10	9	10	0.001 *
No	6	3	7		0	0	1		1	0	2		0	0	4		9	8	10	

* The statistical significance level was set at ≤0.05.

**Table 3:** Descriptive statistics of fatigue levels with participant characteristics.

Participant Characteristics	Current Levels				Desired Levels				Successful Levels				Expected Levels				Importance Levels			
	Median	Q1	Q2	*p*-Value	Median	Q1	Q2	*p*-Value	Median	Q1	Q2	*p*-Value	Median	Q1	Q2	*p*-Value	Median	Q1	Q2	*p*-Value
Gender																				
Male	0	0	8	0.134	0	0	0	0.700	0	0	1	0.753	0	0	0	0.717	10	8	10	0.619
Female	2	0	5		0	0	0		0	0	2		0	0	3		10	9	10	
Age																				
Less Than 30 Years Old	8	6	8	0.000 *	4	0	5	0.000 *	0	0	1	0.023 *	4	4	10	0.000 *	10	7	10	0.000 *
From 30 To 50 Years	0	0	4		0	0	0		0	0	0		0	0	0		10	10	10	
More Than 50 Years	0	0	5		0	0	0		0	0	1		0	0	0		10	8	10	
Martial Status																				
Married	0	0	7	0.000 *	0	0	0	0.000 *	0	0	0	0.000 *	0	0	0	0.000 *	10	9	10	0.000 *
Single	6	2	8		0	0	5		1	0	2		4	0	10		10	6	10	
Widow	5	5	5		5	5	5		4	4	4		3	3	3		6	6	6	
Education Level																				
High School	3	0	8	0.000 *	0	0	0	0.000 *	0	0	1	0.000 *	0	0	3	0.008 *	10	9	10	0.001 *
Bachelor	2	0	8		0	0	4		0	0	2		0	0	5		10	8	10	
Higher Education	0	0	0		0	0	0		0	0	0		0	0	0		10	10	10	
Diploma	0	0	0		0	0	0		0	0	0		0	0	0		10	10	10	
Chronic Illness																				
Yes	0	0	5	0.000 *	0	0	0	0.000 *	0	0	0	0.000 *	0	0	0	0.000 *	10	9	10	0.398
No	3	0	8		0	0	1		0	0	1		0	0	4		10	8	10	

* The statistical significance level was set at ≤0.05.

**Table 4:** Descriptive statistics of distress levels with participant characteristics.

Participant Characteristics	Current Levels				Desired Levels				Successful Levels				Expected Levels				Importance Levels			
	Median	Q1	Q2	*p*-Value	Median	Q1	Q2	*p*-Value	Median	Q1	Q2	*p*-Value	Median	Q1	Q2	*p*-Value	Median	Q1	Q2	*p*-Value
Gender																				
Male	5	0	8	0.205	0	0	0	0.980	0	0	2	0.000 *	0	0	2	0.005 *	10	8	10	0.000 *
Female	5	0	8		0	0	0		0	0	0		0	0	0		10	10	10	
Age																				
Less Than 30 Years Old	8	8	8	0.000 *	5	5	5	0.000 *	0	0	2	0.000 *	4	4	7	0.000 *	10	7	10	0.000 *
From 30 To 50 Years	4	0	7		0	0	0		0	0	0		0	0	0		10	10	10	
More Than 50 Years	5	0	7		0	0	0		0	0	1		0	0	0		10	10	10	
Martial Status																				
Married	5	0	7	0.000 *	0	0	0	0.000 *	0	0	0	0.000 *	0	0	0	0.000 *	10	10	10	0.930
Single	8	8	10		0	0	0		0	0	5		0	0	7		10	7	10	
Widow	0	0	0		5	0	9		4	4	4		0	0	0		10	10	10	
Education Level																				
High School	5	2	8	0.000 *	3	3	3	0.005 *	0	0	0	0.000 *	0	0	2	0.004 *	10	10	10	0.000 *
Bachelor	5	0	7		0	0	0		0	0	2		0	0	5		10	7	10	
Higher Education	0	0	0		0	0	5		0	0	0		0	0	0		10	10	10	
Diploma	10	0	10		0	0	0		0	0	0		0	0	0		10	10	10	
Chronic Illness																				
Yes	5	0	8	0.491	0	0	0	0.001 *	0	0	0	0.000 *	0	0	0	0.000 *	10	10	10	0.002 *
No	5	0	8		0	0	0		0	0	2		0	0	3		10	8	10	

* The statistical significance level was set at ≤0.05.

**Table 5:** Descriptive statistics of daily activity interference levels with participant characteristics.

Participant Characteristics	Current Levels				Desired Levels				Successful Levels				Expected Levels				Importance Levels			
	Median	Q1	Q2	*p*-Value	Median	Q1	Q2	*p*-Value	Median	Q1	Q2	*p*-Value	Median	Q1	Q2	*p*-Value	Median	Q1	Q2	*p*-Value
Gender																				
Male	4	0	8	0.038 *	0	0	0	0.001 *	0	0	0	0.768	0	0	6	0.028 *	10	10	10	0.294
Female	5	2	6		0	0	0		0	0	1		0	0	3		10	9	10	
Age																				
Less Than 30 Years Old	5	0	7	0.002 *	0	0	8	0.000 *	1	0	10	0.000 *	8	4	8	0.000 *	10	10	10	0.000 *
From 30 To 50 Years	3	0	5		0	0	0		0	0	0		0	0	0		10	9	10	
More Than 50 Years	5	0	8		0	0	0		0	0	0		0	0	0		10	10	10	
Martial Status																				
Married	4	0	8	0.066	0	0	0	0.000 *	0	0	0	0.000 *	0	0	0	0.000 *	10	10	10	0.000 *
Single	5	0	7		0	0	0		1	0	10		8	4	8		10	10	10	
Widow	6	6	6		0	0	10		3	3	3		0	0	0		5	5	5	
Education Level																				
High School	4	0	6	0.000 *	4	4	4	0.004 *	0	0	0	0.000 *	0	0	3	0.000 *	10	9	10	0.000 *
Bachelor	5	2	9		0	0	0		0	0	4		5	0	10		10	10	10	
Higher Education	0	0	0		0	0	7		0	0	0		0	0	0		9	9	9	
Diploma	8	0	8		0	0	0		0	0	0		0	0	0		10	10	10	
Chronic Illness																				
Yes	5	2	8	0.000 *	0	0	0	0.002 *	0	0	0	0.042 *	0	0	0	0.000 *	10	9	10	0.125
No	3	0	7		0	0	0		0	0	1		0	0	7		10	10	10	

* The statistical significance level was set at ≤0.05.

## Data Availability

The original contributions presented in the study are included in the article; further inquiries can be directed to the corresponding author.

## Appendix A. The Questionnaire

**We Thank You for Your Agreement to Participate in This Survey:**	**ﻧﺸﻜﺮ ﻟﻜﻢ ﻣﻮاﻓﻘﺘﻜﻢ ﻋﻠﻰ اﻟﻤﺸﺎرﻛﺔ ﻓﻲ ھﺬا اﻻﺳﺘﻄﻼع،**
Perception of periodontitis patients about treatment outcomes: A Cross-Sectional Study in Saudi Arabia.	تصورات مرضى التهاب اللثة المزمن لمخرجات العلاج: دراسة مقطعية في المملكة العربية السعودية.
This assesses four domains relevant to chronic pain populations (pain, fatigue, distress, and interference); through this questionnaire, we will use your answer to check the questionnaire’s validity and publish the Arabic version of PCO, and to evaluate chronic periodontitis using the Arabic version, which contributes to more accessible future research and an assessment of the person-centered concept. It will take you five minutes to complete this questionnaire, and we assure you that all answers will be treated with complete confidentiality and that they have been collected for the purpose of the study only. We assure you that this participation is optional.	الذي يقيم أربعة مجالات ذات صلةبمجموعات الألم المزمن (الألم ، والارهاق ،والضيق ، والتدخل) من خلال هذا الاستبيان ، سوف نستخدم إجابتك للتحقق من صحةالاستبيان ونشر النسخة العربية من PCO ولتقييم التهاب اللثة المزمن باستخدام النسخة العربية. مما يساهم في بحث وتقييممستقبلي يمكن الوصول إليه بسهولة للمفهوم الذي يركز على الشخص. سوف يستغرق إكمالهذا الاستبيان خمس دقائق، ونؤكد لك أن جميع الإجابات ستعامل بسرية تامة وأنه تمجمعها لغرض الدراسة فقط. نؤكد لكم أن هذه المشاركة اختيارية.
I reiterate my thanks to you for your time in participating in this study.	أﻛﺮر ﻟﻚ ﺷﻜﺮي ﻋﻠﻰ وﻗﺘﻚ ﻓﻲ اﻟﻤﺸﺎرﻛﺔ ﻓﻲ ھﺬهاﻟﺪراﺳﺔ.
Researcher/Khaled Al-Khuraiji	اﻟﺒﺎﺣﺚ / ﺧﺎﻟﺪ اﻟﺨﺮﯾﺠﻲ
Many people experience pain, fatigue (i.e., feeling tired), emotional distress (e.g., worries, feeling sad), and interference with daily activities (e.g., not being able to work or do household chores) as a result of their medical condition. We would like to understand how you have been impacted in each of these areas. We would also like to learn more about what you want your treatment to do for you.	العديد من الأشخاص يعانون من الألم ، والارهاق (أي ، الشعور بالتعب) ، والاضطراب العاطفي (على سبيل المثال ، القلق ، الشعور بالحزن) ، والتداخل مع الأنشطة اليومية (على سبيل المثال ، عدم القدرة على العمل أو القيام بأعمال منزلية) كنتيجة لحالتهم الطبية. نود أن نفهم حول ما تريد أن يقدمه لك العلاج. كيفالمجالات. نرغب أيضًا في معرفة المزيد تأثرت في كل من هذه
First, we would like to know your current levels of pain, fatigue, emotional distress, and interference.	أولاً ، نود أن نعرف مستوياتك الحالية من الألم والارهاق والاضطراب العاطفي والتداخل.
On a scale of 0 (none) to 10 (worst imaginable), please indicate your usual level (during the past week) of …	على مقياس من 0 (لا شيء) إلى 10 (أسوأ ما يمكن تخيله) ، يرجى الإشارة إلى مستواك المعتاد (خلال الأسبوع الماضي) من ...
Pain ____	الم _____
Fatigue (Or Tiredness) _____	الارهاق (أو التعب) ______
Emotional Distress _____	الاضطراب العاطفي______
Interference With Daily Activities ______	التداخل في الأنشطة اليومية ______
Now, we would like to learn about your desired levels of pain, fatigue, emotional distress, and interference. In other words, we would like to understand what your ideal treatment outcome would be.	الآن ، نود أن نتعرف على مستوياتك المرغوبة من الألم والإرهاق والاضطراب العاطفي التداخل. بعبارات أخرى ، نود أن نفهم ما ستكون عليه نتيجة العلاج المثالي.
On a scale of 0 (none) to 10 (worst imaginable), please indicate your desired level of …	على مقياس من 0 (لا شيء)إلى 10 (أسوأ ما يمكن تخيله) ، يرجى تحديد المستوى الذي تريده من ...
Pain _____	الم ______
Fatigue (Or Tiredness) _____	الارهاق (أو التعب) ______
Emotional Distress _____	الاضطراب العاطفي______
Interference With Daily Activities _____	التداخل في الأنشطة اليومية ______
Patients understandably want their treatment to result in desired or ideal outcomes like you indicated above. Unfortunately, available treatments do not always produce desired outcomes. Therefore, it is important for us to understand what treatment outcomes you would consider successful.	يرغب المرضى بشكل مفهّم في أن ينتج عن علاجهم نتائج مرغوبة أو مثالية كما أشرت إليها أعلاه. لسوء الحظ ، لا تنتج العلاجات المتوفرة دائمًا النتائج المرجوة. لذلك ، من المهم بالنسبة لنا أن نفهم ما هي نتائج العلاج التي ستعتبرها ناجحة.
On a scale of 0 (none) to 10 (worst imaginable), please indicate the level each of these areas would have to be at for you to consider treatment successful.	على مقياس من 0 (لا شيء) إلى 10 (أسوأ ما يمكن تخيله) ،يرجى الإشارة إلى المستوى الذي يجب أن تكون عليه كل من هذه المجالات حتى تتمكن من اعتبار العلاج ناجحًا.
Pain ____	الم ______
Fatigue (Or Tiredness) _____	الارهاق (أو التعب) ______
Emotional Distress _____	الاضطراب العاطفي______
Interference With Daily Activities _____	التداخل في الأنشطة اليومية ______
Now, we would like to know what you expect your treatment to do for you.	الآن ، نود أن نعرف ما تتوقع أن يقدمه علاجك لك.
On a scale of 0 (none to 10 (worst imaginable), please indicate the levels you expect following treatment.	على مقياس من 0 (لا شيء) إلى 10 (أسوأ ما يمكن تخيله) ، يرجى الإشارة إلى المستويات المتوقعة بعد العلاج.
Pain ____	الم ______
Fatigue (Or Tiredness) ____	الارهاق (أو التعب) ______
Emotional Distress ____	الاضطراب العاطفي______
Interference With Daily Activities _____	التداخل في الأنشطة اليومية ______
Finally, we would like to understand how important it is for you to see improvement in your pain, fatigue, emotional distress, and interference following treatment.	أخيرًا ، نود أن نفهم مدى أهمية أن ترى تحسنًا في الألم ، والارهاق ، والاضطراب العاطفي ، والتداخل بعد العلاج.
On a scale of 0 (not at all important) to 10 (most important), please indicate how important it is for you to see improvement in your…	على مقياس من 0 (ليس مهمًا على الإطلاق) إلى 10 (الأهم) ، يرجى الإشارة إلى مدى أهمية أن ترى تحسنًا في ...
Pain ____	الم ______
Fatigue (Or Tiredness) ____	الارهاق (أو التعب) ______
Emotional Distress ____	الاضطراب العاطفي______
Interference With Daily Activities _____	التداخل في الأنشطة اليومية ______

## References

[1] Listgarten M.A. (1986). Pathogenesis of periodontitis. J. Clin. Periodontol..

[2] Savage A., Eaton K.A., Moles D.R., Needleman I. (2009). A systematic review of definitions of periodontitis and methods that have been used to identify this disease. J. Clin. Periodontol..

[3] Ramseier C.A., Anerud A., Dulac M., Lulic M., Cullinan M.P., Seymour G.J., Faddy M.J., Bürgin W., Schätzle M., Lang N.P. (2017). Natural history of periodontitis: Disease progression and tooth loss over 40 years. J. Clin. Periodontol..

[4] Schätzle M., Löe H., Ramseier C.A., Bürgin W., Ånerud Å., Boysen H., Lang N.P. (2010). Clinical course of chronic periodontitis: Effect of lifelong light smoking (20 years) on loss of attachment and teeth. J. Investig. Clin. Dent..

[5] Chabanski M.B., Gillam D., Bulman J.S., Newman H. (1996). Prevalence of cervical dentine sensitivity in a population of patients referred to a specialist Periodontology Department. J. Clin. Periodontol..

[6] Fiehn N.-E., Larsen T., Christiansen N., Holmstrup P., Schroeder T.V. (2005). Identification of Periodontal Pathogens in Atherosclerotic Vessels. J. Periodontol..

[7] Hegde R., Awan K.H. (2019). Effects of periodontal disease on systemic health. Disease-a-Month.

[8] Graziani F., Karapetsa D., Alonso B., Herrera D. (2000). Nonsurgical and surgical treatment of periodontitis: How many options for one disease?. Periodontology.

[9] Sanz I., Alonso B., Carasol M., Herrera D., Sanz M. (2012). Nonsurgical Treatment of Periodontitis. J. Evid. Based Dent. Pract..

[10] Deas D.E., Moritz A.J., Sagun R.S., Gruwell S.F., Powell C.A. (2016). Scaling and root planing vs. conservative surgery in the treatment of chronic periodontitis. Periodontology 2000.

[11] Slots J., Rams T.E. (1990). Antibiotics in periodontal therapy: Advantages and disadvantages. J. Clin. Periodontol..

[12] Goh V., Hassan F.W., Baharin B., Rosli T.I. (2022). Impact of psychological states on periodontitis severity and oral health-related quality of life. J. Oral Sci..

[13] Jaumet L., Hamdi Z., Julia C., Hercberg S., Touvier M., Bouchard P., Carra M.C., Andreeva V.A. (2023). Periodontitis assessed with a new screening tool and oral health-related quality of life: Cross-sectional findings among general-population adults. Qual. Life Res..

[14] Botelho J., Machado V., Proença L., Bellini D.H., Chambrone L., Alcoforado G., Mendes J.J. (2020). The impact of nonsurgical periodontal treatment on oral health-related quality of life: A systematic review and meta-analysis. Clin. Oral Investig..

[15] Palaiologou A., Kotsakis G.A. (2020). Dentist-Patient Communication of Treatment Outcomes in Periodontal Practice: A Need for Dental Patient–Reported Outcomes. J. Evid. Based Dent. Pract..

[16] Adulyanon S., Sheiham A., Slade G. (1997). Oral impacts on daily performances. Meas. Oral Health Qual. Life.

[17] Mounssif I., Bentivogli V., Rendón A., Gissi D.B., Maiani F., Mazzotti C., Mele M., Sangiorgi M., Zucchelli G., Stefanini M. (2023). Patient-reported outcome measures after periodontal surgery. Clin. Oral Investig..

[18] Needleman I., Almond N., Leow N., Phillips J. (2023). Outcomes of periodontal therapy: Strengthening the relevance of research to patients. A co-created review. Periodontology 2000.

[19] Shah K.S., Xu H., Matsouaka R.A., Bhatt D.L., Heidenreich P.A., Hernandez A.F., Devore A.D., Yancy C.W., Fonarow G.C. (2017). Heart Failure With Preserved, Borderline, and Reduced Ejection Fraction: 5-Year Outcomes. J. Am. Coll. Cardiol..

[20] Al Bush M.M. (2019). An oral cavity profile in illicit- Drug abusers?. J. Indian Soc. Periodontol..

[21] Dirham A.A. (2021). Patient Satisfaction towards Non-Surgical Periodontal Therapy. EC Dent. Sci..

[22] Gautam S., Galgali S.R., Mishra A. (2016). Patients’ Perception Towards Periodontal Therapy: A Cross-Sectional Survey. J. Appl. Dent. Med. Sci..

[23] Faul F., Erdfelder E., Lang A.G., Buchner A. (2007). G* Power 3: A flexible statistical power analysis program for the social, behavioral, and biomedical sciences. Behav. Res. Methods.

[24] Alkhurayji K., Aldakhil S., Alhrabi N., Aljuaid M., Asiri F. (2024). The Translation and Validation of Patient-Centered Outcomes Questionnaire Into Arabic: A Chronic Periodontitis Patient-Based Study. Cureus.

[25] Stutts L.A., Robinson M.E., McCulloch R.C., Banou E., Waxenberg L.B., Gremillion H.A., Staud R. (2009). Patient-centered outcome criteria for successful treatment of facial pain and fibromyalgia. J. Orofac. Pain.

[26] Assiry A.A., Adil A.H., Naing N.N. (2021). Periodontal Disease among Saudi Arabia and South Asian Developing Nations. Int. J. Pharm. Res..

[27] Liu L., Zhang Y., Wu W., Cheng R. (2015). Characteristics of dental care-seeking behavior and related sociodemographic factors in a middle-aged and elderly population in northeast China. BMC Oral. Health.

[28] Saccomanno S., Saran S., Laganà D., Mastrapasqua R.F., Grippaudo C. (2022). Motivation, Perception, and Behavior of the Adult Orthodontic Patient: A Survey Analysis. BioMed Res. Int..

[29] Walther C., Spinler K., Borof K., Kofahl C., Heydecke G., Seedorf U., Beikler T., Terschüren C., Hajek A., Aarabi G. (2022). Evidence from the Hamburg City Health Study—Association between education and periodontitis. BMC Public Health.

[30] Javali M., Betsy J., Al Thobaiti R.S., Alshahrani R., AlQahtani H.A. (2020). Relationship between Malocclusion and Periodontal Disease in Patients Seeking Orthodontic Treatment in Southwestern Saudi Arabia. Saudi J. Med. Med. Sci..

[31] Al-Ahmari J.M., Al-Qarni K.A., Ain T.S., Alkahtani Z.M., Togoo R.A., Mir S. (2021). Factors Affecting Patient’s Satisfaction with the Appearance of Teeth, Gums and Treatments that Improve Aesthetics in Saudi Arabian Sub-Population. J. Evol. Med. Dent. Sci..

[32] Almoudi M.M., Hussein A.S., Abu Hassan M.I., Zain N.M. (2018). A systematic review on antibacterial activity of zinc against Streptococcus mutans. Saudi Dent. J..

[33] Rekhi A., Marya C.M., Oberoi S.S., Nagpal R., Dhingra C., Kataria S. (2016). Periodontal status and oral health-related quality of life in elderly residents of aged care homes in Delhi. Geriatr. Gerontol. Int..

[34] Al-Khabbaz A.K., Al-Shammari K.F., Al-Saleh N.A. (2011). Knowledge about the association between periodontal diseases and diabetes mellitus: Contrasting dentists and physicians. J. Periodontol..

[35] Deng Y., Xiao J., Ma L., Wang C., Wang X., Huang X., Cao Z. (2024). Mitochondrial Dysfunction in Periodontitis and Associated Systemic Diseases: Implications for Pathomechanisms and Therapeutic Strategies. Int. J. Mol. Sci..

[36] Qilichovna A.M. (2024). To Study the Factors That Cause Periodontitis. J. New Century Innov..

[37] Camponogara J.G., de Ferreira T.G.M., Pelissari T.R., Anversa A.M., Moreira C.H.C., Bier C.A.S. (2023). Demographics, smoking status, and systemic health factors associated with apical periodontitis in a Brazilian rural population: A cross-sectional study. Clin. Oral Investig..

